# Harnessing synthetic biology for tetraterpenoid astaxanthin production: Recent advances and challenges

**DOI:** 10.1016/j.synbio.2025.08.005

**Published:** 2025-08-18

**Authors:** Yue Hou, Ailin Guan, Xuefen Fan, Jiufu Qin

**Affiliations:** College of Biomass Science and Engineering, Sichuan University, Chengdu, 610065, China

**Keywords:** Astaxanthin, Microbial cell factories, Metabolic engineering

## Abstract

Astaxanthin, a potent lipid-soluble ketocarotenoid, exists in various stereoisomeric, geometric isomeric, and esterified forms. Its unique molecular configuration and biological activity confer significant advantages for pharmaceutical applications and nutraceutical supplementation. While widely distributed in natural environments, particularly abundant in marine ecosystems, astaxanthin production via conventional methods fails to meet escalating market demands and consumer preference for natural products. Recent synthetic biology advances enable engineered microbial cell factories for astaxanthin production. Through precise genome editing and metabolic reprogramming, these systems offer a sustainable, efficient alternative to traditional synthesis of this high-value antioxidant. In this review, we highlight the latest advancements in constructing artificial cell factories for the production of astaxanthin. Specially, we systematically examine current breakthroughs in synthetic biology-enabled astaxanthin manufacturing, with emphasis on three dimensions: “point” (regulation of key enzymatic activity and expression levels), “line” (pathway-level spatial coordination to balance intermediate flux), and “plane” (system-level metabolic harmonization to address product toxicity and pathway-host metabolic incompatibility). We further discuss critical gaps requiring interdisciplinary innovation to realize the full potential of microbial cell factories in sustainable astaxanthin manufacturing production.

## Graphical abstract

**Figure:**
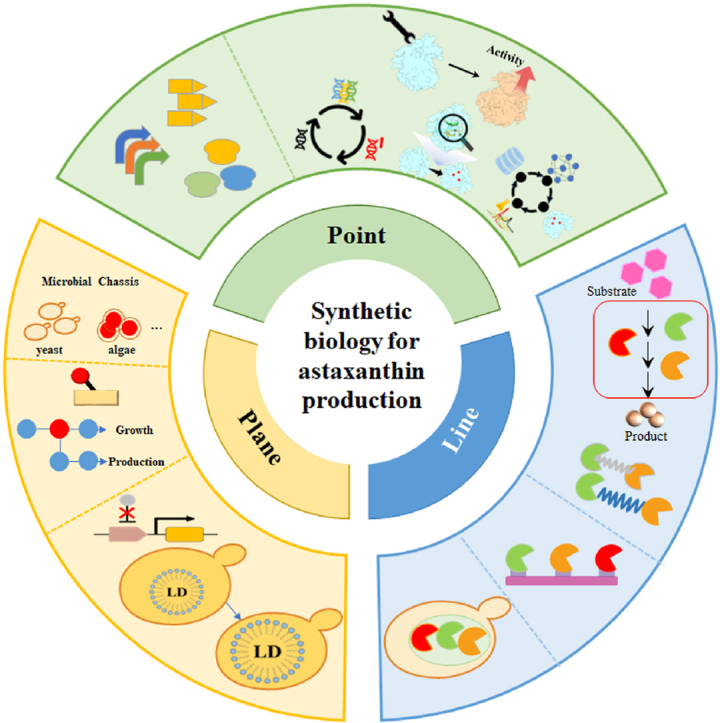

## 1 Introduction

Astaxanthin is a ketocarotenoid with the chemical name 3,3′-dihydroxy-4,4′-diketo-β,β′-carotene, which is naturally abundant in algae (e.g., *Haematococcus pluvialis*), marine organisms (such as shrimp, crab, and salmon), and bird feathers [[1], [2], [3], [4]]. According to reports, the global astaxanthin market is expected to reach $3.4 billion by 2030 [5]. Extensive research has demonstrated its remarkable biological efficacy in immunomodulation, anti-inflammatory mediation, and suppression of tumorigenesis and carcinogenesis, positioning it as a highly promising compound for pharmaceutical and nutraceutical applications [[6], [7], [8]]. However, current commercial production methodologies, including chemical synthesis, natural extraction, and conventional biosynthesis, present significant limitations that constrain their capacity to satisfy escalating market demands. Astaxanthin, obtained via chemical synthesis routes, yields racemic mixtures (3S, 3′S; 3R, 3′R; and 3R, 3′S) with compromised bioactivity and introduces chemical impurities, compromising the product's biological safety [9,10]. Consequently, chemically synthesized astaxanthin is primarily restricted to use in aquaculture and is not permitted for applications in human food or cosmetics under many regulatory frameworks. Given the increasing demand for natural astaxanthin (3S, 3′S) [5], numerous studies have focused on extracting it from biological sources. However, natural extraction from plants, animals, or microorganisms involves technically challenging purification steps and low yields [11]. *Adonis annua* is currently the only higher plant reported to produce astaxanthin, yielding up to 1 % of petal dry weight (DW) [12]. Alternatively, astaxanthin was extracted from marine organisms, where it predominantly exists as fatty acid esters or in free forms [12]. However, astaxanthin derived from these sources faces seasonal supply limitations, technically demanding extraction processes, and high production costs, rendering it unsuitable for large-scale supply.

To date, although microbial fermentation using *H*. *pluvialis* and *Xanthophyllomyces dendrorhous* remains predominant, its application is subject to multiple constraints. *H. pluvialis* requires a two-stage cultivation process, substantially prolonging the production cycle*. X. dendrorhous* exhibits inherently low natural astaxanthin yields, and its genetic regulatory network for carotenoid synthesis remains unclear [13]. While environmental stressors, such as light regulation, nitrogen limitation, enhance astaxanthin accumulation in *H. pluvialis* [[14], [15], [16]], process optimization fails to fundamentally resolve the limitation of extended cultivation timelines. Notably, genetically engineered *X. dendrorhous* achieves yields up to 9.7 mg/g dry cell weight (DCW) [17], yet high-producing strains suffer from reduced efficiency due to growth retardation and intermediate metabolite accumulation. Consequently, the current genetic background of natural producers is unclear, leading to a lack of gene editing tools, as well as the producers remain hindered by protracted cycles and elevated costs, demanding innovative solutions.

With the rapid advancement of synthetic biology, microbial chassis can now be precisely reprogrammed through genome editing and metabolic engineering to construct cell factories for the tailored biosynthesis of high-value chemicals such as vinblastine [18]. This biomanufacturing strategy offers a sustainable and cost-effective alternative to astaxanthin synthesis. However, its implementation is hindered by three major challenges: (1) point-level bottlenecks stemming from the limited availability of high-performance enzymes; (2) line-level incompatibility caused by imbalanced enzymatic reactions; and (3) plane-level incompatibility due to metabolic burden and spatial disorganization. To address the first challenge, *Saccharomyces cerevisiae* was engineered with phylogenetically optimized *crtZ* and *crtW* genes from *Brevundimonas vesicularis* and *Alcaligenes* sp., respectively. Coupled with promoter optimization (e.g., TEF1p), this strategy yielded 4.5 mg/g DCW and 81 mg/L in fed-batch fermentation [19]. For the second challenge, *Escherichia coli* was optimized by fine-tuning CrtZ/CrtW expression ratios and amplifying crtY, alongside co-expression of the groES-groEL chaperone system. This balanced pathway configuration enabled production of 1.18 g/L astaxanthin in fermentation [20]. To overcome system-level constraints, spatial engineering in *Yarrowia lipolytica* was employed by anchoring CrtZ-CrtW fusion proteins to lipid droplets, the endoplasmic reticulum, and peroxisomes. This minimized metabolic crosstalk, reduced intermediate accumulation by 90 %, and leveraged the lipid-rich host physiology to achieve 858 mg/L astaxanthin—a 141-fold improvement over the parental strain [21].

This review systematically examines current breakthroughs in synthetic biology-enabled astaxanthin manufacturing, with emphasis on three dimensions: “point” (enzyme-level optimization), “line” (pathway-level spatial coordination), and “plane” (system-level metabolic harmonization). We further discuss critical gaps requiring interdisciplinary innovation to realize the full potential of microbial cell factories in sustainable astaxanthin manufacturing.

## 2 Diversity of astaxanthin synthesis pathways

**Fig. 1:**
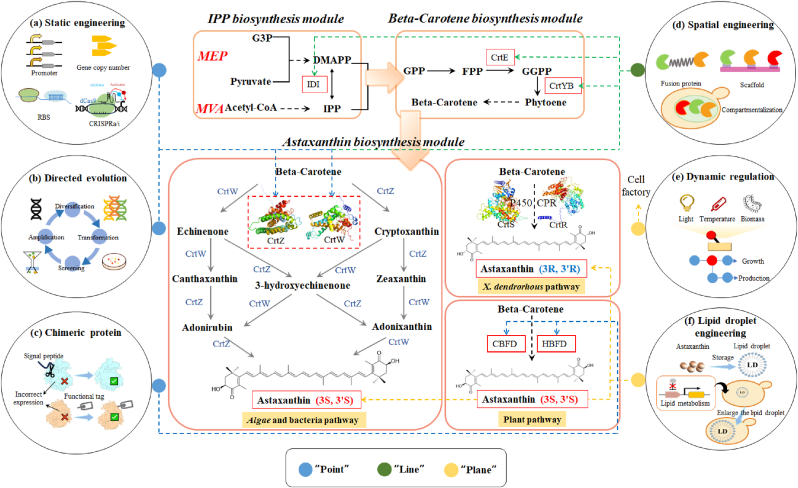
Synthetic biology strategies for enhancing astaxanthin production. (a) Static engineering optimizes pathway flux via RBS engineering, gene copy number tuning (chromosomal/plasmid-based replication), promoter engineering (transcriptional control), and CRISPR activation/interference (CRISPR a/i) for precise gene regulation; (b) Directed evolution improve enzyme catalytic activity and stability; (c) Chimeric protein ensures correct protein expression; (d) Spatial engineering strategies include fusion proteins to channel substrates, scaffold-mediated enzyme colocalization, and compartmentalization to mitigate diffusion limits and substrate inhibition; (e) Dynamic control decouples cell growth from astaxanthin accumulation using inducible systems responsive to temperature, light, biomass signals, or metabolite levels; (f) Lipid droplet engineering enhances intracellular storage capacity. The blue dashed arrows represent “point”. The green dashed arrows represent “Line”. The yellow dashed arrows represent “plane”.

The synthesis pathway of astaxanthin can generally be divided into three main modules: the synthesis of the precursor IPP, the synthesis of β-carotene, and the conversion of astaxanthin (Fig. 1). The precursor synthesis module is a common pathway for the synthesis of all terpenoids, which produces the C5 precursors 5-carbon isomer isopentenyl pyrophosphate (IPP) and dimethylallyl pyrophosphate (DMAPP) through the mevalonate (MVA) or 2-C-methyl-d-erythritol 4-phosphate (MEP) pathway [[22], [23], [24], [25], [26]]. The MVA pathway initiates from acetyl-CoA and culminates in the synthesis of IPP through six enzymatic steps. Its key enzyme, 3-hydroxy-3-methylglutaryl-CoA reductase (HMGR), catalyzes the conversion of HMG-CoA to mevalonate. In contrast, the MEP pathway synthesizes DMAPP through seven enzymatic reactions. Overexpression of key rate-limiting enzymes—1-deoxy-d-xylulose-5-phosphate synthase (DXS), 2-C-methyl-d-erythritol 4-phosphate cytidylyltransferase (MCT), 2-C-methyl-d-erythritol-2,4-cyclodiphosphate synthase (MDS), and 4-hydroxy-3-methylbut-2-enyl-diphosphate synthase (HDR)—in engineered hosts enhances astaxanthin production [[27], [28], [29]]. IPP and DMAPP are interconvertible via isopentenyl diphosphate isomerase (IDI) and serve as precursors for β-carotene synthesis. The MVA pathway requires 2 molecules of NADPH and 3 molecules of ATP, while the MEP pathway requires 3 molecules of NADPH and 3 molecules of ATP.

In the β-carotene synthesis module, IPP and DMAPP further produce C10 compounds geranyl pyrophosphate (GPP), C15 compounds farnesyl pyrophosphate (FPP), and C20 compounds geranylgeranyl pyrophosphate (GGPP), which are important precursors of terpenoids. Through the interaction of GGPP synthase, metabolic flow is competitively diverted from other terpenoids, such as squalene or sterols, to the carotenoid synthesis pathway [30]. Additionally, overexpression of the bifunctional enzyme phytoene synthase/lycopene cyclase (CrtYB), essential for catalyzing phytoene synthesis and lycopene cyclization, enhances astaxanthin production [17,31].

In most organisms, the IPP and β-carotene biosynthesis modules are conserved. However, the conversion of β-carotene to astaxanthin exhibits species-specific differences, broadly categorized into three pathways: algal and bacterial pathway, *X. dendrorhous* pathway, as well as plant pathway (Fig. 1). In the algae *H*. *pluvialis*, β-carotene is converted to 3S,3′S-astaxanthin monoesters and diesters via a two-step hydroxylation reaction and a two-step ketolation reaction catalyzed by the β-carotene ketolase BKT and the β-carotene hydroxylase CrtR-B [32]. The BKT enzymes were localized to different subcellular compartments in *H. pluvialis* [33,34], indicating their essential role in astaxanthin synthesis. In bacteria *Paracoccus carotinifaciens*, the “free” form of 3S,3′S-astaxanthin is synthesized mainly by β-carotene ketolase (CrtW) and β-carotene hydroxylase (CrtZ) [35]. Substrate preferences drive the non-sequential catalysis of β-carotene to astaxanthin by hydroxylases and ketolases in algae and bacteria. In yeast *X. dendrorhous*, this conversion process is catalyzed by the bifunctional enzyme astaxanthin synthase, a cytochrome P450 (CrtS), to produce 3R,3′R-astaxanthin. Expression of CrtS alone is insufficient for astaxanthin production in *X. dendrorhous*, which relies on electron transfer from the FMN domain of cytochrome P450 reductase CrtR [36]. This demonstrates that CrtR is essential for the astaxanthin biosynthesis pathway in *X. dendrorhous*. In plant *A*. *aestivalis*, astaxanthin synthesis involves two key enzymes: carotenoid 4-hydroxy-β-ring-4-dehydrogenase (HBFD) and carotenoid β-ring-4-dehydrogenase (CBFD) [37]. HBFD and CBFD-enzymes add ketone groups and hydroxyl groups to the β-ring of β-carotene, respectively. Their substrate specificity results in an ordered reaction sequence for astaxanthin biosynthesis. It is noteworthy that the above synthetic pathways are synthesized only by the *H. pluvialis* in the 3S-3′S conformation, which exhibits superior immunomodulatory activity in vitro [38].

## 3 Microbial engineering strategies for astaxanthin synthesis

### 3.1 Point—enzyme-level optimization

In bacteria, fungi and algae, the astaxanthin biosynthetic pathway is notably complex, comprising two hydroxylation steps, two ketolation steps, and involving seven metabolic intermediates. The conversion of β-carotene to astaxanthin is catalyzed by β-carotene ketolase and β-carotene hydroxylase, the two most critical enzymes in the biosynthetic pathway. However, the relatively low activity of critical enzymes results in a lower final yield of astaxanthin [32,[35], [36], [37]]. It is imperative to enhance both the expression levels and catalytic efficiency of these two pivotal enzymes. Recent advancements in metabolic engineering and protein engineering offer promising strategies to address these challenges (Fig. 1). Besides, Table 1 summarizes representative examples of astaxanthin biosynthesis, including reported titers and the corresponding engineering strategies employed in strain development.

**Table 1:** Microbial cell factories for astaxanthin production.

Host	Key strategies	Scale	Yield/Titer	Ref.
*E.coli*	Utilizing β-carotene hydroxylases with different substrate preferences.	5-L fermenter	1.82 g/L	[77]
*E.coli*	Screening of exogenous ketolase and hydroxylase and balanced expression.	Shake- flask cultivation	7.4 ± 0.3 mg/g DCW	[39]
*E.coli*	Using λ-Red recombination technology, integrate the genes encoding the astaxanthin biosynthesis pathway into the chromosome of *E. coli*.	Shake- flask cultivation	1.4 mg/g	[40]
*E.coli*	Optimize the relative expression of β-carotene ketolase and hydroxylase using RBS of different strengths.	Bioreactor	320 mg/L	[41]
*E.coli*	Coordinating the expression of β-carotene ketolase and hydroxylase and adding molecular chaperones.	5-L fermenter	1180 mg/L	[20]
*E.coli*	Multi- enzyme assembly using a mimic PKS enzyme assembly line (mPKSeal) strategy.	Shake- flask cultivation	16.9 mg/g DCW	[55]
*E.coli*	Fusion of hydroxylase and ketonase and localization to membranes.	Shake- flask cultivation	/	[57]
*E.coli*	Complementarily expressing the morphology-/membrane-related genes (*lpp* and *bamB*) and the oxidative stress-related genes (*uspE* and *yggE*)	Shake- flask cultivation	11.92 mg/g DCW	[67]
*E.coli*	Introduce *CrtX* to glycosylate astaxanthin.	Shake- flask cultivation	4.82 mg/L	[69]
*S. cerevisiae*	Screening for exogenous ketolase and hydroxylase and replacing strong promoters.	5-L bioreactor(4 % (w/v) glucose)	81.0 mg/L	[19]
*S. cerevisiae*	Directed coevolution of β-carotene ketolase and hydroxylase, and temperature-responsive regulation.	5-L bioreactor	235 mg/L	[47]
*S. cerevisiae*	Directed evolution of fusion of β-carotene hydroxylase and ketolase.	Shake- flask cultivation	1.87 mg/g DCW	[48]
*S. cerevisiae*	Expression of exogenous ketolase and hydroxylase, deletion of *CSS1*.	5-L bioreactor	217.9 mg/L	[78]
*S. cerevisiae*	Deletion of *HRD1*, down-regulation of *OPI3*, overexpression of *Pdr3*.	Shake- flask cultivation	12.26 mg/g DCW	[79]
*S. cerevisiae*	Deletion of *opi3* and *hrd1* moderately upregulates lipid synthesis.	5-L bioreactor	446.4 mg/L	[64]
*Y. lipolytica*	Modular engineering pathways and fusion of key genes	100-L bioreactor	812.3 mg/L	[80]
*Y. lipolytica*	Fine-tuning transcriptional expression of ketolase and hydroxylase and modular assembly.	5-L bioreactor	41.3 mg/g DCW, 3300 mg/L	[53]
*Y. lipolytica*	Localization of the fusion enzyme CrtW-Z to subcellular organelles.	3-L bioreactor	453 mg/L	[21]
*C. glutamicum*	Improved precursor supply and balanced expression of terminal genes ketolase and hydroxylase.	2-L bioreactor	103 mg/L	[68]

[c] The symbol “/” indicates that the article does not specify the yield.

Static regulatory strategy by designing and modifying genetically coded elements can significantly increase the expression level of the enzyme and thus enhance product synthesis (Fig. 1a). The following are the three main regulatory strategies: optimization of gene copy number, fine tuning of translation initiation rate and modulation of promoter activity. Starting at the level of gene replication, optimizing copy number to balance the metabolic flow is a direct way to improve astaxanthin synthesis. Qian et al. [39] found that increased hydroxylase activity is necessary for astaxanthin production, and that integration of two copy numbers of *crtZ* (from *Pantoea ananatis*) into the chromosome of *E. coli* significantly promoted astaxanthin synthesis and increased astaxanthin production by approximate 35 %. While increasing the copy number of the *crtZ* and *crtW* genes, it is also necessary to consider whether the protein is correctly folded. In addition, it is supposed that hydroxylase and ketolase compete for the same substrate, and that only a balanced expression of these enzymes can ensure the complete conversion of β-carotene to astaxanthin [40]. Therefore, the optimal ratio of these enzymes can be finely tuned at the translational level by adjusting their relative translation initiation rates. For example, RBS libraries were constructed for the ketolase and hydroxylase genes, encompassing a range of 50- to 140-fold differences in relative RBS strength, to facilitate the random optimization of strains. Following this optimization, astaxanthin concentration increased by up to fivefold [41]. Moreover, at the transcriptional level, enhancing the expression of astaxanthin synthesizing genes by regulating promoter activity is also an effective strategy. For instance, by upregulating the hydroxylase gene promoter activity to regulate the hydroxylation level and replacing the original promoter FBA1p with the stronger TEF1p, the hydroxylase transcription level increased by 33.5 times and astaxanthin production increased by 30.4 % under 4 % glucose conditions [19]. Optimizing the ratio of β-carotene hydroxylase and ketolase expression by tuning the regulatory element (e.g., promoter, RBS) strength is crucial. However, library construction and screening costs grow exponentially with increasing gene number, making this approach prohibitively costly in time and resources [42]. An alternative strategy is to divide related genes into modules that are independently regulated by different promoters to improve efficiency.

Moreover, approaches such as exogenous enzyme screening, protein modification, and protein engineering represent effective strategies for enhancing the performance of key enzymes. Firstly, by combining heterologous hydroxylases and ketolases from 30 species, the highest astaxanthin yield (3.1 mg/g DCW) was achieved in *S. cerevisiae* expressing *crtW* from *Brevundimonas* and *crtZ* from *Alcaligenes*. Analysis revealed that CrtW plays a more critical role than CrtZ in astaxanthin biosynthesis, with its evolutionary distance correlating with catalytic efficiency [19]. Bacterial CrtWs exhibit broader substrate promiscuity than algal orthologs, enabling multi-step conversion toward higher astaxanthin production. Although CrtZ and CrtW catalyze β-carotene to astaxanthin conversion through complex reactions, the optimal reaction order for their co-catalysis remains unresolved [43,44].

It has been reported that natural ketolase and hydroxylase are typically membrane proteins, and their soluble expression and correct folding are crucial for the conversion of intermediates to astaxanthin. For this purpose, researchers have used protein modification tools based on gene fusion technology, such as co-expression with molecular chaperones, expression of secreted proteins, and fusion proteins as strategies to improve the solubility and folding properties of proteins [45] (Fig. 1c). The *E. coli* chaperone protein GroEL and its cochaperone GroES mediate the ATP-dependent folding of newly translated proteins and improved the correct expression of key enzymes through *groES-groEL* operon modulation strategy, which resulted in an increase in astaxanthin production by 23–45 % [20]. N-terminal fusion of OmpF and C-terminal fusion of TrxA to trCrBKT (from *Chlamydomonas reinhardtii*) resulted in a significant increase in astaxanthin yield [29]. Besides, the *A*. *aestivalis*-derived carotenoid β-ring 4-dehydrogenase CBFD and carotenoid 4-hydroxy-β-ring 4-dehydrogenase HBFD could not achieve catalytic activity in microorganisms due to the presence of chloroplast transit peptides, and CBFD remained active only after N-terminal truncation. The small ubiquitin-like modified protein SUMO, which is used as a tag and molecular chaperone, was further added to promote the correct folding and structural stability of the proteins and regulate the expression of CBFD and HBFD [46].

Furthermore, directed evolution—an approach that accelerates the natural evolutionary process of proteins by increasing the mutation rate—has proven to be an effective strategy for enhancing the catalytic activity of heterologous enzymes (Fig. 1b). Zhou et al. constructed mutant libraries of *ObktM* and *OcrtZ* (from *H. pluvialis*) via error-prone PCR. The optimal mutant OcrtZM1^L288R^ and ObktM29^H165R/V264D/F298Y/M1T/N188D/L271R^ were obtained. The *OcrtZM1* and *ObktM29* were co-integrated into the yeast genome, increasing astaxanthin production by 39 %. Subsequent analysis revealed that the ObktM29 ^M1T^ shifted the translation start site to the 8th amino acid codon, truncating the N-terminus to enhance β-carotene ketolase activity [47]. Alternatively, changing MnCl_2_ concentration to control mutagenesis efficiency enabled successful engineering of fusion enzyme CrtW (from *B*. *vesicularis* DC263) and CrtZ (from *Agrobacterium aurantiacum*). The combination mutant L95S/I206L increased astaxanthin yield by 1.23- and 1.38-fold versus single mutants. Homology models suggest that hydrogen bonding between the mutant and the substrate molecule is significantly enhanced, which facilitates the formation of carbonyl and hydroxyl groups [48]. Currently, the low catalytic activity of rate-limiting enzymes remains the key bottleneck. Screening and combination of enzyme sources, as well as enhancing enzyme activity, constitute effective strategies for optimizing astaxanthin pathways.

### 3.2 Line—pathway-level spatial coordination

Conventional single-enzyme optimization often falls short in addressing mass transfer limitations and intermediate-induced feedback inhibition inherent in complex terpene biosynthetic pathways. In contrast, spatial organization strategies—such as multienzyme complexes, synthetic enzyme scaffolds, and reaction microcompartments—have shown promising potential to overcome these challenges [[49], [50], [51]] (Fig. 1d). The accumulation of intermediates during biosynthesis can cause by-product formation, feedback inhibition, and cytotoxicity, underscoring the importance of their efficient conversion. For instance, fusion of CrtZ and CrtW from *B*. *vesicularis* using a (GGGGS)_2_ flexible linker effectively reduced the accumulation of intermediate metabolites and enhanced astaxanthin production. Compared to the control, the engineered fusion enzyme resulted in a 14-fold reduction in canthaxanthin levels, a 7-fold reduction in zeaxanthin levels, and a 1.6-fold increase in astaxanthin content [48]. It is worth noting that the fusion mode of CrtZ and CrtW and the choice of linker length may affect the catalytic efficiency [52]. Additionally, short peptide tags (RIAD/RIDD) enabled scaffold-free enzyme complex assembly, elevating astaxanthin yield from 1.9 to 2.9 mg/g DCW (12.3–20.9 mg/L) [53,54].

Natural biocatalytic systems often form organized multi-enzyme complexes, enzyme molecular scaffolds, or reaction microchambers to effectively promote the transfer of substrates in multi-step metabolic reactions. Inspired by this, researchers have developed a variety of protein docking strategies to mimic and optimize this efficient process (Fig. 1d). Type I modular polyketide synthases (PKSs) are assemblies of multiple enzymes capable of synthesizing a diverse array of polyketide products. Sun et al. [55] utilized the docking domains (DDs) of type I *cis*-AT PKS as connecting medium to develop a strategy called mimic PKS enzyme assembly line (mPKSeal) for multi-enzyme assembly. They sequentially assembled the cytosolic enzyme IDI and the membrane-associated enzymes CrtE and CrtB into a multienzyme complex, resulting in a 2.4-fold increase in astaxanthin production. The dosage of DDs requires optimization, as excess DDs may impair the catalytic efficiency of membrane proteins. Compared with direct fusion constructs, the multi-enzyme complexes assembled via the above strategy exhibit enhanced catalytic efficiency. Notably, RIAD-RIDD outperforms mPKSeal in carotenoid production, primarily due to differences in the proportion of the enzyme assembly [55].

Since astaxanthin and its synthetic intermediates lycopene, β-carotene, etc., are hydrophobic long-chain terpenoid compounds, they usually accumulate in membrane compartments in prokaryotes, but the mechanism of their synthesis and accumulation is not yet fully understood [56]. To utilize these enzymes more efficiently, compartmentalization engineering has been proposed as a solution, aiming to shorten the catalytic distance between enzyme and substrate (Fig. 1d). The ketolase and hydroxylase as well as their membrane-bound substrates may have three localization configurations: directly localized to the membrane, localized to the membrane after being linked, or only linked but not localized [57]. By simulating these three configurations, the experimental results showed that when the two enzymes were linked by a flexible linker and localized to the membrane after being fused with the GlpF protein, the enzyme localization effect was optimal, and astaxanthin production increased by 215.4 % [57]. After the two enzymes are fixed on the membrane, lower mobility may reduce catalytic efficiency. Targeting astaxanthin-synthesis enzymes to key intermediate production sites enables efficient substrate utilization and reduces intermediate accumulation. This compartmentalization strategy is also exemplified by the membrane-targeted expression of tag-fused trCrBKT in *E. coli*, as previously described. In eukaryotes such as *S. cerevisiae* and *Y. lipolytica*, the biosynthesis of the intermediate β-carotene occurs mainly in the endoplasmic reticulum (ER) and further aggregates in lipid bodies (LB) or is stored in peroxisome, making it difficult for cytoplasmic enzymes to efficiently convert it to astaxanthin. By targeting the astaxanthin synthesis pathway to subcellular organelles LB, ER, and peroxisomes, not only the conversion of β-carotene to astaxanthin accelerated, but also the accumulation of ketocarotenoid intermediates was significantly reduced. When the enzyme targeted all three subcellular organelles simultaneously, astaxanthin yield was further enhanced to 139.4 mg/L, which was a 4.82-fold increase over the original yield. Subsequent fed-batch fermentation of the engineered strain ultimately yielded 858 mg/L of astaxanthin, representing a 141-fold increase compared to the initial strain [21]. The enhanced astaxanthin compartmentalization in engineered cell factories, particularly in eukaryotes with superior cellular complexity over prokaryotes, constitutes an effective strategy for high-yield production. While β-carotene with lipid-rich, predominantly localizes to the ER and LB, astaxanthin distribution varies depending on its esterification state [34,58].

In addition, eliminating competitive pathways is also an effective strategy for enhancing astaxanthin biosynthesis. Astaxanthin biosynthesis competes with lipid metabolism for key precursors. In *X*. *dendrorhous*, ergosterol biosynthesis competes with astaxanthin for acetyl-CoA. This metabolic conflict was exploited by deleting sterol desaturase genes (*CYP61*), redirecting flux toward astaxanthin synthesis and increasing yield by 1.4-fold [59]. Similarly, Kildegaard et al. enhanced β-carotene production through downregulation of the competitive squalene synthase (*SQS1*) [60]. In microalgae, fatty acid and triacylglycerol biosynthesis pathways compete with astaxanthin for pyruvate, with evidence suggesting potential cross-pathway regulatory mechanisms [61,62].

### 3.3 Plane—system-level metabolic harmonization

In the application of engineered metabolic pathways, resource competition between endogenous mechanisms of the host cell and exogenous gene expression leads to host growth defects and reduces the synthesis efficiency and cellular robustness of the target product [63]. Therefore, in-depth revelation of the transport and storage mechanisms of astaxanthin is crucial to further improve the production capacity of astaxanthin. In terms of storage, the accumulation space of the product can be increased through lipid droplet engineering and membrane engineering, or its toxicity can be reduced by modifying the product's properties (such as reducing hydrophobicity), thereby improving the product's stability and accumulation efficiency (Fig. 1f). For example, research identified *opi3* and *hrd1*, genes associated with lipid metabolism, as potential engineering targets. In the strain with double deletion of *hrd1* and *opi3*, the astaxanthin storage ratio in lipid droplets (LDs) increased from 0.7 % to 4.5 %, while the astaxanthin ratio in the cell membrane decreased to 91.1 %. Experimental results indicated that moderate, rather than excessive, enhancement of lipid synthesis can promote astaxanthin production by expanding intracellular storage capacity [64].

Besides, membrane engineering enhances the storage capacity of intracellular products by altering the composition of cell membranes. This strategy was implemented for β-carotene synthesis by remodeling *E. coli* membrane morphology and rewiring lipid biosynthesis pathways [65], coupled with the construction of an artificial membrane vesicle transport system (AMVTS) [66]. Combined down-regulation of the outer membrane protein genes *lpp* and *bamB*, along with the cell shape-related protein gene *rodZ*, resulted in elongated and enlarged cells with higher reactive oxygen species (ROS) levels, leading to a 58 % increase in astaxanthin content [67]. Membrane engineering has also been applied to shorten the distance between astaxanthin synthase and its substrate to increase astaxanthin yield [57].

Limited reports on astaxanthin high-density fermentation suggest that the accumulation of astaxanthin, which belongs to the carotenoid family, leads to growth inhibition of the strain. In addition to increasing intracellular storage space, the cytotoxicity caused by product accumulation can also be reduced by increasing the solubility of the substrate. The glycosylation of many hydrophobic compounds (e.g., lipids and terpenes) in nature is catalyzed by glycosyltransferases, resulting in derivatives with better water solubility. The glycosyltransferase encoded by the crtX gene uses UDP-glucose as a sugar donor to transfer sugar molecules to the hydroxyl groups of carotenoids, a process that results in structural changes that can improve water solubility, photostability, and bioactivity. The process of astaxanthin glycosylation involves the transfer of glucose molecules from UDP-glucose to the hydroxyl group of the astaxanthin β-ionone ring to produce astaxanthin β-d-diglucoside, which has improved solubility and bioavailability compared to unmodified astaxanthin. Glycosylated astaxanthin has been successfully produced in *E. coli*, *Y. lipolytica*, and *Corynebacterium glutamicum* [68,69].

Additionally, the dynamic regulation strategy achieves precise regulation of intracellular metabolic levels by optimizing gene expression, thereby promoting the synthesis of target metabolites while ensuring the supply of precursor substances required for cell growth, thereby maintaining a balance between growth and production (Fig. 1e). Therefore, zhou et al. replaced the *GAL4* gene in *S. cerevisiae*, which had been knocked out of the *GAL80* gene (encoding GAL4 inhibitor), with the temperature-sensitive *Gal4M9* variant. This engineering yielded a yeast cell factory regulated by a thermal-responsive promoter, thereby decoupling cell growth from astaxanthin synthesis. Maintain a higher temperature in the early stage of fermentation to promote cell growth; in the middle and late stages of the logarithmic growth phase, gradually lower the temperature to stimulate the accumulation of astaxanthin [47]. Of course, the compartmentalization strategy mentioned in section 3.2 also contributes to metabolic harmonization.

## 4 Challenges and perspectives

Despite advances in metabolic and protein engineering, chemical synthesis remains the primary source of astaxanthin due to the low yields and high costs of biosynthesis. To bridge this gap, a hierarchical strategy addressing “point-line-plane” challenges is essential.

At the “point” level, enhancing catalytic elements like CrtZ and CrtW is critical. Traditional methods such as static engineering (e.g., codon/promoter optimization) and directed evolution have improved enzyme activity. Protein engineering of astaxanthin synthase remains scarce in the literature, due to insufficient structural characterization data and the absence of robust high-throughput screening methodologies. Emerging artificial intelligence (AI) tools like AlphaFold [70,71] now enable rational protein design, exemplified by its use in modeling aminase structures to classify cytidine deaminases more accurately [72]. In *S*. *cerevisiae* astaxanthin biosynthesis studies, AlphaFold-assisted analysis revealed that mutations of CrtZ-CrtW fusion enzyme significantly enhanced hydrogen bonding at the substrate-active site interface, stabilizing substrate-enzyme binding and improving catalytic activity [48]. Concurrently, structural simulation of the rate-limiting enzyme PaCrtZ (from *P*. *ananatis*) guided alanine substitution to expand the substrate access channel, increasing astaxanthin yield by 39 % through catalytic efficiency enhancement [73]. These advances accelerate the shift from random mutagenesis to precision enzyme engineering. For “line” level regulation, balancing metabolic and energy fluxes is key. While classical Design-Build-Test-Learn (DBTL) cycles are resource-intensive [51], synthetic biology tools like CRISPR/Cas9 [74] for genome editing, combined with cofactor engineering [75], enable precise pathway control. Li et al. employed CRISPR-mediated screening to identify *opi3* and *hrd1* as engineering targets modulating lipid metabolism. Moderate enhancement of lipid biosynthesis redirects metabolic flux, altering more precursors into the astaxanthin synthesis pathway to increase yield in *S*. *cerevisiae* [64]. Machine learning (ML) further optimizes microbial factories by predicting strain behavior and refining metabolic networks [76]. The “plane” level challenge—product cytotoxicity and storage limitations—requires spatial solutions. Expanding lipid droplets, subcellular compartmentalization, and artificial transport systems (e.g., artificial membrane vesicle transport system for β-carotene transport) mitigate toxicity and enhance storage [66].

Overall, astaxanthin's high antioxidant activity and demand in food, nutraceuticals, and cosmetics underscore its commercial potential. The microbial engineering strategies—point-line-plane dimensions—enhance both carbon metabolic flux and astaxanthin storage capacity. Among these approaches, fine tuning astaxanthin synthase activity and expanding subcellular storage compartments demonstrate superior efficacy over other methods. Critically, combinatorial implementation of these strategies exhibits greater potential than singular interventions. However, the commercialization of astaxanthin biosynthesis currently faces multiple technical challenges: persistent genetic instability in engineered strains and the lack of efficient genetic manipulation tools for natural microorganisms and non-conventional yeasts (such as *Y*. *lipolytica*). Future efforts should not only overcome the above technical obstacles but also integrate synthetic biology to optimize yields, reduce costs, and resolve transport bottlenecks. Furthermore, while GRAS-certified chassis (e.g., *S. cerevisiae*, *Y. lipolytica*, and *C. glutamicum*) offer regulatory and consumer advantages for astaxanthin biosynthesis, diverse national regulations for market application remain. Therefore, eliminating these regulatory barriers is key to unlocking astaxanthin's market potential. By combining AI-guided enzyme design, dynamic flux control, and compartmentalized biosynthesis, microbial cell factories could rival chemical production, unlocking sustainable astaxanthin supply at scale.

## CRediT authorship contribution statement

**Yue Hou:** Writing – review & editing, Writing – original draft, Visualization, Validation, Methodology, Investigation, Data curation. **Ailin Guan:** Writing – review & editing, Writing – original draft, Validation, Investigation. **Xuefen Fan:** Investigation, Writing – review & editing. **Jiufu Qin:** Writing – review & editing, Writing – original draft, Visualization, Supervision, Project administration, Investigation, Funding acquisition, Conceptualization.

## Declaration of competing interest

The authors declare that they have no known competing financial interests or personal relationships that could have appeared to influence the work reported in this paper.

## References

[1] Yabuzaki J. (2017). Carotenoids database: structures, chemical fingerprints and distribution among organisms. Database.

[2] Honda M., Kageyama H., Hibino T., Sowa T., Kawashima Y. (2020). Efficient and environmentally friendly method for carotenoid extraction from *Paracoccus carotinifaciens* utilizing naturally occurring *Z*-isomerization-accelerating catalysts. Process Biochem.

[3] Zhou D., Yang X., Wang H., Jiang Y., Jiang W., Zhang W. (2023). Biosynthesis of astaxanthin by using industrial yeast. Biofuel Bioprod Biorefining.

[4] Yamashita E., Misawa N. (2021). Carotenoids: biosynthetic and biofunctional approaches.

[5] Acheampong A., Li L., Elsherbiny S.M., Wu Y., Swallah M.S., Bondzie-Quaye P. (2024). A crosswalk on the genetic and conventional strategies for enhancing astaxanthin production in *Haematococcus pluvialis*. Crit Rev Biotechnol.

[6] Fan Q., Chen Z., Wu Y., Zhu J., Yu Z. (2021). Study on the enhancement of immune function of astaxanthin from *Haematococcus pluvialis*. Foods.

[7] Chang M.X., Xiong F. (2020). Astaxanthin and its effects in inflammatory responses and inflammation-associated diseases: recent advances and future directions. Molecules.

[8] Ren P., Yu X., Yue H., Tang Q., Wang Y., Xue C. (2023). Dietary supplementation with astaxanthin enhances anti-tumor immune response and aids the enhancement of molecularly targeted therapy for hepatocellular carcinoma. Food Funct.

[9] Yu B., Ma T., Nawaz M., Chen H., Zheng H. (2025). Advances in metabolic engineering for the accumulation of astaxanthin biosynthesis. Mol Biotechnol.

[10] Kumar S., Kumar R., Diksha Kumari A., Panwar A. (2022). Astaxanthin: a super antioxidant from microalgae and its therapeutic potential. J Basic Microbiol.

[11] Wan X., Zhou X.R., Moncalian G., Su L., Chen W.C., Zhu H.Z. (2021). Reprogramming microorganisms for the biosynthesis of astaxanthin via metabolic engineering. Prog Lipid Res.

[12] Nishida Y., Berg P., Shakersain B., Hecht K., Takikawa A., Tao R. (2023). Astaxanthin: past, present, and future. Mar Drugs.

[13] Bellora N., Moliné M., David-Palma M., Coelho M.A., Hittinger C.T., Sampaio J.P. (2016). Comparative genomics provides new insights into the diversity, physiology, and sexuality of the only industrially exploited tremellomycete: *Phaffia rhodozyma*. BMC Genom.

[14] Hong M.-E., Hwang S.K., Chang W.S., Kim B.W., Lee J., Sim S.J. (2015). Enhanced autotrophic astaxanthin production from *Haematococcus pluvialis* under high temperature via heat stress-driven Haber–Weiss reaction. Appl Microbiol Biotechnol.

[15] Christian D., Zhang J., Sawdon A.J., Peng C.-A. (2018). Enhanced astaxanthin accumulation in *Haematococcus pluvialis* using high carbon dioxide concentration and light illumination. Bioresour Technol.

[16] Ding W., Zhao Y., Xu J.-W., Zhao P., Li T., Ma H. (2018). Melatonin: a multifunctional molecule that triggers defense responses against high light and nitrogen starvation stress in *Haematococcus pluvialis*. J Agric Food Chem.

[17] Gassel S., Schewe H., Schmidt I., Schrader J., Sandmann G. (2013). Multiple improvement of astaxanthin biosynthesis in *Xanthophyllomyces dendrorhous* by a combination of conventional mutagenesis and metabolic pathway engineering. Biotechnol Lett.

[18] Zhang J., Hansen L.G., Gudich O., Viehrig K., Lassen L.M.M., Schrübbers L. (2022). A microbial supply chain for production of the anti-cancer drug vinblastine. Nature.

[19] Wang R., Gu X., Yao M., Pan C., Liu H., Xiao W. (2017). Engineering of β-carotene hydroxylase and ketolase for astaxanthin overproduction in *Saccharomyces cerevisiae*. Front Chem Sci Eng.

[20] Gong Z., Wang H., Tang J., Bi C., Li Q., Zhang X. (2020). Coordinated expression of astaxanthin biosynthesis genes for improved astaxanthin production in *Escherichia coli*. J Agric Food Chem.

[21] Ma Y., Li J., Huang S., Stephanopoulos G. (2021). Targeting pathway expression to subcellular organelles improves astaxanthin synthesis in *Yarrowia lipolytica*. Metab Eng.

[22] Berthelot K., Estevez Y., Deffieux A., Peruch F. (2012). Isopentenyl diphosphate isomerase: a checkpoint to isoprenoid biosynthesis. Biochimie.

[23] Chang W., Song H., Liu H., Lu P. (2013). Current development in isoprenoid precursor biosynthesis and regulation. Curr Opin Chem Biol.

[24] Gwak Y., Hwang Y., Wang B., Kim M., Jeong J., Lee C.-G. (2014). Comparative analyses of lipidomes and transcriptomes reveal a concerted action of multiple defensive systems against photooxidative stress in *Haematococcus pluvialis*. J Exp Bot.

[25] Brown M.S., Goldstein J.L. (1980). Multivalent feedback regulation of HMG CoA reductase, a control mechanism coordinating isoprenoid synthesis and cell growth. J Lipid Res.

[26] Banerjee A., Sharkey T.D. (2014). Methylerythritol 4-phosphate (MEP) pathway metabolic regulation. Nat Prod Rep.

[27] Li Q., Fan F., Gao X., Yang C., Bi C., Tang J. (2017). Balanced activation of IspG and IspH to eliminate MEP intermediate accumulation and improve isoprenoids production in *Escherichia coli*. Metab Eng.

[28] Liu Y.-H., Alimujiang A., Wang X., Luo S.-W., Balamurugan S., Yang W.-D. (2019). Ethanol induced jasmonate pathway promotes astaxanthin hyperaccumulation in *Haematococcus pluvialis*. Bioresour Technol.

[29] Park S.Y., Binkley R.M., Kim W.J., Lee M.H., Lee S.Y. (2018). Metabolic engineering of *Escherichia coli* for high-level astaxanthin production with high productivity. Metab Eng.

[30] Zhao J., Li Q., Sun T., Zhu X., Xu H., Tang J. (2013). Engineering central metabolic modules of *Escherichia coli* for improving β-carotene production. Metab Eng.

[31] Zhou P., Xie W., Li A., Wang F., Yao Z., Bian Q. (2017). Alleviation of metabolic bottleneck by combinatorial engineering enhanced astaxanthin synthesis in *Saccharomyces cerevisiae*. Enzym Microb Technol.

[32] Vidhyavathi R., Venkatachalam L., Sarada R., Ravishankar G.A. (2008). Regulation of carotenoid biosynthetic genes expression and carotenoid accumulation in the green alga *Haematococcus pluvialis* under nutrient stress conditions. J Exp Bot.

[33] Grünewald K., Hirschberg J., Hagen C. (2001). Ketocarotenoid biosynthesis outside of Plastids in the unicellular green Alga *Haematococcus pluvialis*. J Biol Chem.

[34] Chen G., Wang B., Han D., Sommerfeld M., Lu Y., Chen F. (2015). Molecular mechanisms of the coordination between astaxanthin and fatty acid biosynthesis in *Haematococcus pluvialis* (Chlorophyceae). Plant J.

[35] Scaife M.A., Burja A.M., Wright P.C. (2009). Characterization of cyanobacterial β‐carotene ketolase and hydroxylase genes in *Escherichia coli*, and their application for astaxanthin biosynthesis. Biotechnol Bioeng.

[36] Alcaíno J., Fuentealba M., Cabrera R., Baeza M., Cifuentes V. (2012). Modeling the interfacial interactions between CrtS and CrtR from *Xanthophyllomyces dendrorhous*, a P450 system involved in astaxanthin production. J Agric Food Chem.

[37] Cunningham F.X. Jr., Gantt E. (2011). Elucidation of the pathway to astaxanthin in the flowers of *Adonis aestivalis*. Plant Cell.

[38] Sun W., Xing L., Lin H., Leng K., Zhai Y., Liu X. (2016). Assessment and comparison of in vitro immunoregulatory activity of three astaxanthin stereoisomers. J Ocean Univ China.

[39] Lu Q., Bu Y.-F., Liu J.-Z. (2017). Metabolic engineering of *Escherichia coli* for producing astaxanthin as the predominant carotenoid. Mar Drugs.

[40] Lemuth K., Steuer K., Albermann C. (2011). Engineering of a plasmid-free *Escherichia coli* strain for improved in vivo biosynthesis of astaxanthin. Microb Cell Fact.

[41] Zhang C., Seow V.Y., Chen X., Too H.-P. (2018). Multidimensional heuristic process for high-yield production of astaxanthin and fragrance molecules in *Escherichia coli*. Nat Commun.

[42] Smanski M.J., Bhatia S., Zhao D., Park Y., B A., Woodruff L., Giannoukos G. (2014). Functional optimization of gene clusters by combinatorial design and assembly. Nat Biotechnol.

[43] Zhou P., Ye L., Xie W., Lv X., Yu H. (2015). Highly efficient biosynthesis of astaxanthin in *Saccharomyces cerevisiae* by integration and tuning of algal crtZ and bkt. Appl Microbiol Biotechnol.

[44] Scaife M.A., Ma C.A., Ninlayarn T., Wright P.C., Armenta R.E. (2012). Comparative analysis of β-Carotene hydroxylase genes for astaxanthin biosynthesis. J Nat Prod.

[45] Zhou Q., Huang D., Yang H., Hong Z., Wang C. (2024). Improvement of carotenoids' production by increasing the activity of beta-carotene ketolase with different strategies. Microorganisms.

[46] Chen J., Zhang R., Zhang G., Liu Z., Jiang H., Mao X. (2023). Heterologous expression of the plant-derived astaxanthin biosynthesis pathway in *Yarrowia lipolytica* for glycosylated astaxanthin production. J Agric Food Chem.

[47] Zhou P., Li M., Shen B., Yao Z., Bian Q., Ye L. (2019). Directed coevolution of β-Carotene ketolase and hydroxylase and its application in temperature-regulated biosynthesis of astaxanthin. J Agric Food Chem.

[48] Ding Y.-W., Lu C.-Z., Zheng Y., Ma H.-Z., Jin J., Jia B. (2023). Directed evolution of the fusion enzyme for improving astaxanthin biosynthesis in *Saccharomyces cerevisiae*. Synth Syst Biotechnol.

[49] Dutta S., Whicher J.R., Hansen D.A., Hale W.A., Chemler J.A., Congdon G.R. (2014). Structure of a modular polyketide synthase. Nature.

[50] Kerfeld C.A., Aussignargues C., Zarzycki J., Cai F., Sutter M. (2018). Bacterial microcompartments. Nat Rev Microbiol.

[51] Volk M.J., Tran V.G., Tan S.-I., Mishra S., Fatma Z., Boob A. (2023). Metabolic engineering: methodologies and applications. Chem Rev.

[52] Henke N.A., Wendisch V.F. (2019). Improved astaxanthin production with *Corynebacterium glutamicum* by application of a membrane fusion protein. Mar Drugs.

[53] Zhu H.-Z., Jiang S., Wu J.-J., Zhou X.-R., Liu P.-Y., Huang F.-H. (2022). Production of high levels of 3 *S* ,3′ *S* -Astaxanthin in *Yarrowia lipolytica* via iterative metabolic engineering. J Agric Food Chem.

[54] Kang W., Ma T., Liu M., Qu J., Liu Z., Zhang H. (2019). Modular enzyme assembly for enhanced cascade biocatalysis and metabolic flux. Nat Commun.

[55] Sun X., Yuan Y., Chen Q., Nie S., Guo J., Ou Z. (2022). Metabolic pathway assembly using docking domains from type I cis-AT polyketide synthases. Nat Commun.

[56] Doshi R., Nguyen T., Chang G. (2013). Transporter-mediated biofuel secretion. Proc Natl Acad Sci U S A.

[57] Ye L., Zhu X., Wu T., Wang W., Zhao D., Bi C. (2018). Optimizing the localization of astaxanthin enzymes for improved productivity. Biotechnol Biofuels.

[58] Mascia M., Girolomoni L., P, Alcocer M.J., Bargigia I., Perozeni F., Cazzaniga S. (2017). Functional analysis of photosynthetic pigment binding complexes in the green alga *Haematococcus pluvialis* reveals distribution of astaxanthin in photosystems. Sci Rep.

[59] Yamamoto K., Hara K.Y., Morita T., Nishimura A., Sasaki D., Ishii J. (2016). Enhancement of astaxanthin production in *Xanthophyllomyces dendrorhous* by efficient method for the complete deletion of genes. Microb Cell Fact.

[60] Kildegaard K.R., Adiego-Pérez B., Doménech Belda D., Khangura J.K., Holkenbrink C., Borodina I. (2017). Engineering of *Yarrowia lipolytica* for production of astaxanthin. Synth Syst Biotechnol.

[61] Zhang Y., Kawasaki Y., Oshita K., Takaoka M., Minami D., Inoue G. (2021). Economic assessment of biogas purification systems for removal of both H2S and siloxane from biogas. Renew Energy.

[62] Zhang Y., Ye Y., Ding W., Mao X., Li Y., Gerken H. (2020). Astaxanthin is ketolated from zeaxanthin independent of fatty acid synthesis in *Chromochloris zofingiensis*. Plant Physiol.

[63] Mao J., Zhang H., Chen Y., Wei L., Liu J., Nielsen J. (2024). Relieving metabolic burden to improve robustness and bioproduction by industrial microorganisms. Biotechnol Adv.

[64] Li M., Zhou P., Chen M., Yu H., Ye L. (2022). Spatiotemporal regulation of astaxanthin synthesis in *S. cerevisiae*. ACS Synth Biol.

[65] Wu T., Ye L., Zhao D., Li S., Li Q., Zhang B. (2017). Membrane engineering - a novel strategy to enhance the production and accumulation of β-carotene in *Escherichia coli*. Metab Eng.

[66] Wu T., Li S., Ye L., Zha D., Fan Q., Li Q. (2019). Engineering an artificial membrane vesicle trafficking system 2 (AMVTS) for the excretion of β-carotene in *Escherichia coli*. ACS Synth Biol.

[67] Lu Q., Liu J.-Z. (2019). Enhanced astaxanthin production in *Escherichia coli* via morphology and oxidative stress engineering. J Agric Food Chem.

[68] Göttl V.L., Meyer F., Schmitt I., Persicke M., Peters-Wendisch P., Wendisch V.F. (2024). Enhancing astaxanthin biosynthesis and pathway expansion towards glycosylated C40 carotenoids by *Corynebacterium glutamicum*. Sci Rep.

[69] Chen X., Lim X., Bouin A., Lautier T., Zhang C. (2021). High-level de novo biosynthesis of glycosylated zeaxanthin and astaxanthin in *Escherichia coli*. Bioresour Bioprocess.

[70] Varadi M., Anyango S., Deshpande M., Nair S., Natassia C., Yordanova G. (2022). AlphaFold protein structure database: massively expanding the structural coverage of protein-sequence space with high-accuracy models. Nucleic Acids Res.

[71] Baek M., DiMaio F., Anishchenko I., Dauparas J., Ovchinnikov S., Lee G.R. (2021). Accurate prediction of protein structures and interactions using a three-track neural network. Science.

[72] Huang J., Lin Q., Fei H., He Z., Xu H., Li Y. (2023). Discovery of deaminase functions by structure-based protein clustering. Cell.

[73] Wang N., Guo W., Yang C., Wang K., Li G., Liu D. (2024). Improved astaxanthin synthesis in *Komagataella phaffii* through β-Carotene ketolase screening and β-Carotene hydroxylase mutagenesis. ACS Food Sci Technol.

[74] Thean D.G.L., Chu H.Y., Fong J.H.C., Chan B.K.C., Zhou P., Kwok C.C.S. (2022). Machine learning-coupled combinatorial mutagenesis enables resource-efficient engineering of CRISPR-Cas9 genome editor activities. Nat Commun.

[75] Chen R., Gao J., Yu W., Chen X., Zhai X., Chen Y. (2022). Engineering cofactor supply and recycling to drive phenolic acid biosynthesis in yeast. Nat Chem Biol.

[76] Vavricka C.J., Takahashi S., Watanabe N., Takenaka M., Matsuda M., Yoshida T. (2022). Machine learning discovery of missing links that mediate alternative branches to plant alkaloids. Nat Commun.

[77] Zhang M., Gong Z., Tang J., Lu F., Li Q., Zhang X. (2022). Improving astaxanthin production in *Escherichia coli* by co-utilizing CrtZ enzymes with different substrate preference. Microb Cell Fact.

[78] Jin J., Wang Y., Yao M., Gu X., Li B., Liu H. (2018). Astaxanthin overproduction in yeast by strain engineering and new gene target uncovering. Biotechnol Biofuels.

[79] Mao S., Yu H., Ye L. (2023). Enhanced astaxanthin production in *S. cerevisiae* by combinatorial engineering of gene targets outside the synthetic pathway. Biochem Eng J.

[80] Yuzbasheva E.Y., Taratynova M.O., Fedyaeva I.M., Dementev D.A., Korobov V.S., Fedorov A.S. (2023). Large-scale bioproduction of natural astaxanthin in *Yarrowia lipolytica*. Bioresour Technol Rep.

